# Editorial: Mucosal barrier in teleost fish: physical, biochemical, and immune nature

**DOI:** 10.3389/fimmu.2024.1349071

**Published:** 2024-01-16

**Authors:** Xiuzhen Sheng, Li Lin, Roy Ambli Dalmo, Jianmin Ye

**Affiliations:** ^1^ Laboratory of Pathology and Immunology of Aquatic Animals, Key Laboratory of Mariculture, Ministry of Education (KLMME), Ocean University of China, Qingdao, China; ^2^ College of Animal Sciences and Technology, Zhongkai University of Agriculture and Engineering, Guangzhou, China; ^3^ Faculty of Biosciences, Fisheries and Economics, UiT Arctic University of Norway, Tromsø, Norway; ^4^ School of Life Sciences, South China Normal University, Guangzhou, China

**Keywords:** mucosal barrier, teleost fish, physical, biochemical, immune

The mucosal surfaces of fish play a crucial role in protecting against diseases by acting as barriers to potentially harmful agents like bacteria, viruses, fungi and parasites. The advances in research on the physical, biochemical, and immune nature of these mucosal barriers in fish will increase our understanding of the protective mechanisms involved in defending against pathogens and maintaining the body’s homeostasis. This Research Topic includes six original research articles.

In teleost fish, three immunoglobulin isotypes, namely IgM, IgD and IgT, have been identified (1, 2). Among these, IgM is the principal Ig involved in both humoral and mucosal immunity, while IgT is considered to be a mucosal Ig although it also is involved in systemic immune responses (3, 4). The IgT gene and function remains still unknown in numerous teleost species. Chan et al. identified the secretory IgT (sIgT) gene of Asian seabass (*Lates calcarifer*) and assessed its expression in healthy and nervous necrosis virus (NNV)-infected fish, in both mucosal and non-mucosal tissues. They recombinantly expressed IgT and its CH2-CH4 domains, and developed polyclonal antibodies against IgT (CH2-CH4) to enable detection of IgT and IgT^+^ cells. IgT^+^ cells were found in gills and intestine, and the highest levels of sIgT mRNA were detected in mucosal and lymphoid tissues such as the gills, intestine, skin and head kidney. Following infection trough an immersion experiment, IgT expression was upregulated in mucosal tissues and head kidney, and mucosal IgT, but not IgM, was significantly increased in the gills and intestine. These findings suggest that IgT plays a crucial role in protecting the mucosal surface, similar to mammalian IgA, by strengthening the immune barrier against pathogen infection.

Three articles investigated the effects of supplementing the basal diet with an antibacterial peptide, probiotic, and pungent spices on the mucosal barrier and health of fish. Because of the insufficient supply of fish meal/oil in aquaculture, a replacement by soybean meal (SM) and vegetable oils has become an economic viable and attractive approach. However, the question of whether immune additives, such as antibacterial peptide, yeast, or plant additives, can reduce the side effects caused by excessive use of replacement nutrients in farmed fish, such as apoptosis and inflammation (5, 6), tissue damage (7), and fat accumulation in enterocytes (8), remains unresolved. To mitigate SM induced disorder, Huo et al. developed the nano antibacterial peptide CMCS-gcIFN-20H (C-I20) to supplement the diet for mandarin fish (*Siniperca chuatsi*). They found a 160 mg kg^-1^ C-I20 supplement improved growth performance and reduced feed conversion ratio, while appropriate goblet cell numbers, mucin thickness, and villus length were maintained, revealing the improvement of intestinal mucus and epithelial barrier. It also reduced tissue injury/inflammation in the liver, trunk kidney, head kidney and spleen, thereby alleviating the negative effects of SM feed. The other two articles were performed using gilthead seabream (*Sparus aurata*) as the model species. Sanahuja et al. explored the effects of supplementing a low fish meal-based diet with *Debaryomyces hansenii* (1.1% and 2.2%), a yeast that naturally occurs in carnivorous fish and has been shown to improve fish health, as a probiotic. A transcriptomic study of fish’s skin tissue was performed after 70 days of feeding, and the secreted mucus associated biomarkers and defensive capacity was evaluated. They found *D. hansenii* administration strongly influenced immune-related processes, particularly those involved in B- and T-cell regulation, suggesting a modulated immune response. It also promoted the skin barrier function by upregulating anchoring junction genes, which reinforced the physical defense against potential skin damage. The increased defensive capacity was proved by an increase in protective function of skin mucus against bacterial pathogens. Their results indicated probiotic could enhance the skin’s physical barrier and mucus defensive capacity while maintaining immune and metabolic homeostasis in the skin mucosa. Furthermore, Ruiz et al. investigated the effects of supplementing a fish oil-reduced diet containing poultry fat with a combination of pungent spices (capsicum, black pepper, ginger, and cinnamaldehyde). They found that the supplementation with 0.1% pungent spices enhanced growth performance, reduced feed conversion rate, decreased perivisceral fat index, and increased the DHA/EPA ratio in the fillet. Particularly, the diet lowered fat deposits in the visceral cavity, liver and intestine, and prevented intestinal inflammation or physiological disorders maintaining intestinal barrier integrity, thereby improving the fish’s health status and condition.

Zhang et al. provided evidence supporting that gene loss and co-option of Toll-like receptors (TLRs) play a role in the immune adaptation of male seahorses during pregnancy. TLRs, as pattern recognition receptors (PRR), are important in recognizing antigens from bacteria, viruses, yeast and parasites together with damage associated molecular patterns (DAMPS). TLR proteins are involved in immune activation and play crucial roles in mammalian pregnancy by balancing host resistance and immune tolerance in the uterus and placenta (9–12). In seahorses with enclosed brood pouches and sophisticated placentas, the paternal immune resistance to the fetus is a critical checkpoint. Zhang et al. discovered that all syngnathid species lost three vertebrate-conserved TLRs (TLR1, TLR2, and TLR9), while the TLR paralog genes (TLR18, TLR25, and TLR21) were highly expressed in the placenta (a folded inner pseudostratified columnar epithelium) inside the seahorse brood pouch and changed dynamically during the breeding cycle and in response to specific pathogenic antigens, revealing a possible immunological checkpoint. This immunological adaptive mechanism caused by TLR gene family evolution provided new insight into the role of TLRs in mucosal barrier. The research society should, in a common effort, try to identify as many of the TLR ligands as possible, and to study their function with respect to changing environment. This may create knowledge that can be used beyond their pure immunological function.

Finally, the research by Zhao et al. presented a simple feasible approach for preparing cell suspension, enriched by leucocytes, from the intestinal mucosa of zebrafish (*Danio rerio*), by separating the mucosal villi from the muscle layer. They obtained intestinal immune cell suspension with higher expression of both innate and adaptive immune genes. This cell suspension was enriched in immune-related genes and pathways, as well as PRR signaling and cytokine-cytokine receptor interaction. The enriched cell preparations can be used to study intestinal inflammatory responses, where inflammatory Bowel disease in humans is an apparent candidate. The use of e.g., zebra fish as a model has received high level of interest for translational research. Their results also gain a better understanding of teleost intestinal cellular immunity, and the molecular composition and regulation of intestinal barrier function.

Indeed, the findings from all of these studies contribute to our understanding of the mechanisms that regulate mucosal barrier integrity, mucosal immune response in aquatic animals and as checkpoints. They also facilitate further research aimed at improving mucosal health and, therefore, the well-being of aquacultured animals.

## Author contributions

XS: Writing – original draft, Writing – review & editing. LL: Writing – original draft, Writing – review & editing. RD: Writing – original draft, Writing – review & editing. JY: Writing – original draft, Writing – review & editing.
